# Study on the Therapeutic Benefit on Lactoferrin in Patients with Colorectal Cancer Receiving Chemotherapy

**DOI:** 10.1155/2014/184278

**Published:** 2014-10-28

**Authors:** Tarek M. Moastafa, Alaa El-Din Elsayed El-Sissy, Gehan K. El-Saeed, Mai Salah El-Din Koura

**Affiliations:** ^1^Faculty of Pharmacy, Tanta University, Tanta, Egypt; ^2^Pharmacology Department, Faculty of Pharmacy, Tanta University, Tanta, Egypt; ^3^Faculty of Medicine, Menoufiya University, Shibin el-Kawm, Egypt; ^4^Hospital of Menoufiya University, Shibin el-Kawm, Egypt

## Abstract

A double-blinded parallel randomized controlled clinical trial was conducted on two groups of colorectal cancer patients to study the therapeutic benefit of orally administered bovine lactoferrin (bLF) on colorectal cancer patients having age ranges from 20 to 71 years and who received 5-fluorouracil and leucovorin calcium. Test group (15 patients) received oral bLF 250 mg/day beside chemotherapy for three months. Control group (15 patients) received chemotherapy only. Serum lactoferrin (LF), serum glutathione-s-transferase enzyme (GST), interferon gamma (INF-*γ*), tumor marker carcinoembryonic antigen (CEA), renal function tests, hepatic function tests, and complete blood count were measured for both groups before and at the end of the trial. Although, there was a significant effect of oral bLF (250 mg/day) that indicated a significant improvement in mean percent of change of all parameters 3 months after treatment, there was no significant difference between results of patients in the test group and patients in the control group after treatment. This result suggests that oral bLF has significant therapeutic effect on colorectal cancer patients. Our study suggests that daily administration of bLF showed a clinically beneficial effect to colorectal cancer patients with better disease prognosis but that needs further looking into.

## 1. Introduction

Lactoferrin (Lf) is an iron-binding glycoprotein of the transferrin family that is expressed and secreted by glandular cells, such as milk, saliva, tears, and mucous secretions [1]. It is also found in the neutrophils from which it is released into infected tissues and blood during the inflammatory process [1]. Initially described as an iron-binding molecule with bacteriostatic properties, Lf is now known to be a multifunctional or multitasking protein [1]. It is a major component of the innate immune system of mammals. Its protective effects range from direct antimicrobial activities against a large panel of microorganisms, including bacteria, viruses, fungi, and parasites, to anti-inflammatory and anticancer activities [1]. Lf has multiple activities relying not only on its capacity to bind iron, but also to interact with molecular and cellular components of both host and pathogens. Lf can bind and sequester lipopolysaccharide (LPS), thus, preventing proinflammatory pathway activation, sepsis, and tissue damage. However, Lf-bound LPS may retain the capacity to induce cell activation [2].

Lactoferrinis thought to cause a therapeutic revolution, because it was proven in the last few years that LF could be used in the treatment of many diseases such as hepatitis C virus [3], osteoporosis [4], diabetic foot ulcers [5], and cancer [6].

A large number of studies prove that oral lactoferrin has therapeutic effect on cancer, but most of these studies were conducted on animals [7, 8]. Also there were former preclinical studies that approved the chemoprevention effect of LF on colorectal cancer [9, 10] and its ability to decrease chemotherapy related side effects [11]. In our clinical trial, we use oral bLF as an adjuvant therapy for metastatic colorectal cancer patients who were receiving chemotherapy in order to evaluate the role of lactoferrin on the outcome of colorectal cancer and its role in minimizing chemotherapy induced toxicity to enhance quality of life.

Colorectal cancer is a cancer of the large intestine (colon) and rectal cancer is a cancer of the last several inches of the colon. Together, they're often referred to as colorectal cancers [12].

Many symptoms have been described, with the main ones being rectal bleeding, diarrhea, or constipation (change in bowel habit), loss of weight, abdominal pain, and anemia. However, these symptoms are also common with benign conditions [12]. There is no test available for use in primary care that provides a sufficient discrimination to help in referral decisions although; primary care investigations sometimes include fecal occult blood testing and estimation of hemoglobin [13].

5-fluorouracil (5-FU) and leucovorin are the most commonly used regimen in the treatment of colorectal cancer [14]. However, the prognosis is usually poor, so in our study, we aimed to use bLF as adjuvant therapy to the previously mentioned chemotherapy regimen in order to evaluate the outcome of bLF implication in such cancerous patients.

## 2. Methodology

### 2.1. Patients and Method

A double-blinded parallel randomized controlled clinical trial was conducted on 30 colorectal cancer patients in the tumor institution of Menoufiya University receiving 5-FU and calcium leucovorin every 28 days. Patients were divided into two groups; one of them was the test group in which patients received oral bLF 250 mg/day for plus 5-FU and calcium leucovorin for 3 months. The other group was the control group in which patients received chemotherapy only for the same period.

(i) The endpoints of this clinical trial are as follows.
 The primary endpoint is a quantitative endpoint by measuring serum LF levels of all patients in the two groups 3 months after treatment.
 The secondary end point is enhancing quality of life for patients in test group which appear in improving renal and hepatic functions tests and also appear in relieving mucositis.
(ii) Parameters measured to determine oral bLF efficacy were
  (1) serum LF
  (2) serum GST enzyme
  (3) serum INF-*γ*
  (4) CEA
(iii) Parameters measured to determine oral bLF safety were
  (1) renal functions tests [BUN-serum creatinine]
  (2) hepatic functions tests [ALT-AST]
  (3) hematological toxicity [CBC]

### 2.2. Sample Size Calculations

The sample size is the number of patients or other experimental units included in a study [15]. These calculations are particularly of interest in the design of randomized controlled trials (RCTs) [15].

Simplest formula for a continuous outcome and equal sample sizes in both groups, assuming: alpha = 0.05 and power = 0.80 (beta = 0.20)

 
*n* = the sample size in each of the groups
 
*μ*1 = population mean in treatment Group 1
 
*μ*2 = population mean in treatment Group 2
 
*μ*1 − *μ*2 = the difference the investigator wishes to detect
 
*σ*2 = population variance (SD)
 
*a* = conventional multiplier for alpha = 0.05
 
*b* = conventional multiplier for power = 0.80
  (1) $$\begin{array}{c} \frac{n=2\left[{\left(a+b\right)}^{2}\sigma^{2}\right]}{{\left(\mu 1-\mu 2\right)}^{2}}. \end{array}$$

The significance level alpha is chosen at 0.05; one should enter the value 1.96 for *a* in the formula. Similarly, when beta is chosen at 0.20, the value 0.842 should be filled in for *b* in the formula [15].

The sample size calculation is based on using the population variance of a given outcome variable that is estimated by means of the standard deviation (SD) in case of a continuous outcome. Because the variance is usually an unknown quantity, investigators often use an estimate obtained from a pilot study or use information from a previously performed study [15]. In our study, we use SD information from a previously performed study that investigated the effect of orally administered bovine lactoferrin on the growth of adenomatous colorectal polyps [16]. In this clinical study, results indicated that serum hLF levels mean ± SD changed by 25.43 ± 19.35 ng/mL in the 3.0 g bLF group for 12 months [16].

As our clinical trial was carried out using oral bLF 250 mg per day for only 3 months, we calculate SD of LF group from the data of the former clinical study.

 LF dose in the former clinical study is 3000 mg per day resulted in SD of 19.35.
 LF dose in our clinical study is 250 mg per day so SD should be 1.6.
 LF SD in our clinical study is 1.6 in 12 months.

So, the calculated SD of LF in our clinical study for 3 months is 0.403

*μ*1 − *μ*2: the smallest effect of interest is the minimal difference between the studied groups that the investigator wishes to detect [15]. In our clinical study, we consider a difference in serum LF of 0.45 ng/mL 3 months after treatment between the treated and the control groups.

Calculate *n*:

(2) $$\begin{array}{c} n=\frac{2\left[{\left(1.96+.8942\right)}^{2}*0.403^{2}\right]}{{\left[0.45\right]}^{2}}=12.59. \end{array}$$

This means that a sample size of 13 subjects per group is needed but 15 patients were participating in each group.

Blood samples were collected from patients in the two groups at the beginning of the trial period and 3 months after treatment. Samples were divided into two parts; one of them was used as a whole blood sample for CBC and differential blood count testing using ADVIA 120 Hematology System (ADVIA kits, Siemens, Germany). Also to measure is CEA through Enzyme Linked Fluorescent assay (ELFA) using mini VIDAS (VIDAS kit, BioMérieux, France). The other part was separated in the centrifuge 2000 rps (Jouan centrifuge) to obtain serum samples for evaluation of (BUN), serum creatinine, aspartate aminotransferase (AST), and alanine aminotransferase (ALT), all of which were analyzed spectrophotometry using Beckman Synchron CX9 Pro Chemistry Analyzer (Beckman kits, Beckman, California, United States), serum LF by ELISA using TECAN ELISA reader (ELISA kit Assaypro Company, Saint Charles, United States, Catalog Number EL2011-1), serum GST enzyme by ELISA using TECAN ELISA reader (ELISA kit Cusabio, United States, Company Catalog Number: CSB-E09032h), and INF-*γ* by ELISA using TECAN ELISA reader (ELISA kit Assaypro Company, Saint Charles, United States, Catalog Number: EI1023-1).

## 3. Statistical Analysis

The results were statistically analyzed by dependent sample-*T* test for each group to compare between patients results in the same group before and after 3 months to determine the effect of bLF oral administration on test group. Independent sample-*T* test was also used to compare between results of patients in the test group and results of patients in the control group before and after 3 months. Statistical analysis was done using SPSS computer software version 20. Results are represented as mean ± SD using significance level of *P* ≤ 0.05.

## 4. Ethical Standards

All patients in the trial were informed and, therefore, the trial has been performed in accordance with the ethical standards of the tumor institution of Menoufiya University.

## 5. Results

Patients who participated in this clinical trial were metastatic colorectal cancer patients (stage 4).

*Characteristics of Patients Who Participated in This Clinical Study (Table 1).*

- Two patients who had hypertension were on an oral drug that is a combination of captopril and hydrochlorothiazide.
- Two patients who had diabetes mellitus type 2 one of them on oral hypoglycemic and the other on insulin.
- Two patients who were on warfarin due to old deep vein thrombosis (DVT).

### 5.1. Biochemical Results

No significant difference was found in BUN, serum creatinine, AST, ALT, serum LF,GST enzyme, INF-*γ*, WBCs count, platelet count, CEA, CA19.9, RBCs count, neutrophil count, and Hb level of patients in the test and control groups before beginning of the trial at significance level (*P* ≤ 0.05) (Table 2).

A comparison between patients parameters values in test and control groups 3 months after treatment indicates that no significant difference in mean values of serum creatinine, AST, ALT, serum LF, serum GST enzyme, INF-*γ*, WBCs count, platelet count, CEA, RBCs count, neutrophil count, and Hb level of patients in test and control groups except for BUN values in test group were significantly decreased (*P* = 0.048) and INF-*γ* values were significantly increased (*P* = 0.041) at significance level (*P* ≤ 0.05) (Table 3).

Bovine LF administration resulted in significant increase in serum LF (*P* = 0.003), serum GST enzyme (*P* = 0.001), INF-*γ* (*P* = 0.001), WBCs count (*P* = 0.004), platelet count (*P* = 0.001), RBCs count (*P* = 0.001), neutrophil count (*P* = 0.001), and Hb level (*P* = 0.001); also bLF administration resulted in significant decrease in BUN (*P* = 0.001), creatinine (*P* = 0.05), ALT (*P* = 0.033), AST (*P* = 0.003), and CEA (*P* = 0.033) as compared to all parameters values of patients in control group at significance level (*P* ≤ 0.05) (Table 4).

Mean percent of change of main parameters (serum LF, serum GST enzyme, and INF-*γ*) after than before treatment among the studied patients indicate significant improvement in patients who received oral bLF 3 months after treatment at significance level (*P* ≤ 0.05) (Table 5).

Mean percent of change of the remaining parameters (CEA, CBC, renal function tests, and hepatic function tests) after than before treatment among the studied patients indicate significant improvement in patients who received oral bLF 3 months after treatment at significance level (*P* ≤ 0.05) (Table 6).

### 5.2. Clinical Parameters of Metastatic Colorectal Cancer Patients

#### 5.2.1. CT Scan

About 90% of metastatic colorectal cancer patients in the trial underwent a surgery, either partial or total colectomy.

(i) CT scan reports for all patients in the test group and the control group before surgery: circumferential soft tissue thickening was seen in different parts of the colon and rectum with the largest bulk of the mass seen measuring from 49∗61∗30 mm in all various dimensions up to 7∗4.5 cm in its axial dimension.
(ii) CT scan reports for all patients in the test group and control group after surgery and before the trial: no significant time interval changes.
(iii) CT scan reports for all patients in the test and control groups after the trial period 3 months:
   no significant time interval changes
   no de novo changes are noted
   no recurrent masses.

#### 5.2.2. Biopsy

- Biopsy reports for all patients in the test and control groups before surgery showed mucinous adenocarcinoma at different stages and grades that differ from patient to another.

#### 5.2.3. Incidence and Severity of Oral Mucositis

- Before receiving bLF: patients in the test group and control group had moderate to severe oral mucositis after every chemotherapy cycle.
- After receiving bLF: patients in the test group had less severe mucositis than patients in the control group after every chemotherapy cycle indicated by an enhanced ability to swallow.

#### 5.2.4. Recurrence of Infection (Fever Incidence and Degree)

- Before receiving bLF: patients in the test group and control group hadhigh rate of infection recurrence with high degree of fever.
- After receiving bLF: patients in test group had a lesser rate of infection recurrence and had less incidence of fever than patients in control group.

## 6. Discussion

**Figure 1:**
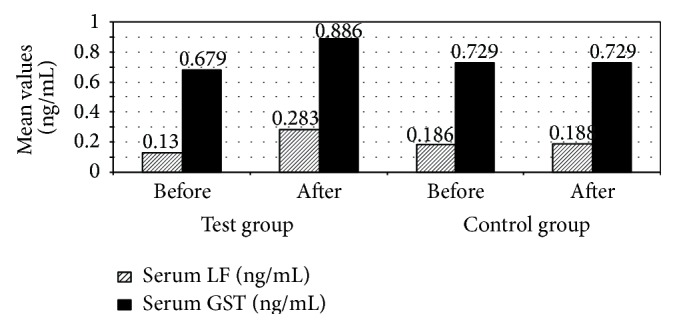
Mean serum lactoferrin (LF) and serum glutathione-s-transferase (GST) among the studied patients with colorectal cancer receiving chemotherapy (treated with recombinant human lactoferrin and not).

This parallel randomized controlled clinical trial aimed to evaluate the role of oral bLF on the outcome of colorectal cancer and its role in minimizing chemotherapy induced toxicity to enhance patient's quality of life. The results of this trial indicate that oral bLF made a significant increase in serum LF levels of patients in the test group 3 months after treatment compared to patients in the control group (*P* ≤ 0.05).  Figure 1 indicates that mean serum LF increased from 0.13 ng/mL before treatment to 0.283 ng/mL after 3 months of treatment in test group compared to mean serum LF of the control group which had very small increase from 0.186 ng/mL before treatment to 0.188 ng/mL after 3 months of treatment. This may be due to the bLF-mediated induction of serum human lactoferrin (hLF) levels via activation of neutrophils [16]. Lactoferrin is an immunomodulator agent that may support the proliferation, differentiation, and activation of immune cells and strengthen the immune response [17].

It is well known that inflammation initiates sporadic colorectal cancer because most intratumoral immune cells are recruited after the tumor is formed and so, in this case, chronic inflammation does not precede but follows tumor development. However, after a tumor forms, the localized inflammatory microenvironment can promote the accumulation of additional mutations and epigenetic changes. Activated inflammatory cells produce reactive oxygen species (ROS) and reactive nitrogen intermediates that can induce DNA damage and mutation [18]. Due to LF iron binding properties and interactions with target cells and molecules, it can influence immune system cells and cells involved in the inflammation reaction [17]. Lactoferrin acts as an anti-inflammatory factor, due to its antimicrobial activity and capability of binding components of bacterial cell walls (LPS) or their receptors; lactoferrin may prevent the development of inflammation and subsequent tissue damage caused by the release of pro-inflammatory cytokines and reactive oxygen species [17]. The protective effect of lactoferrin manifests in a reduced production of some proinflammatory cytokines and an increased amount of anti-inflammatory cytokines [17]. Iron is essential as a catalyst for the production of reactive oxygen species. Therefore, lactoferrin can diminish the harmful influence of reactive oxygen species produced by leukocytes at the sites of inflammation [17].

**Figure 2:**
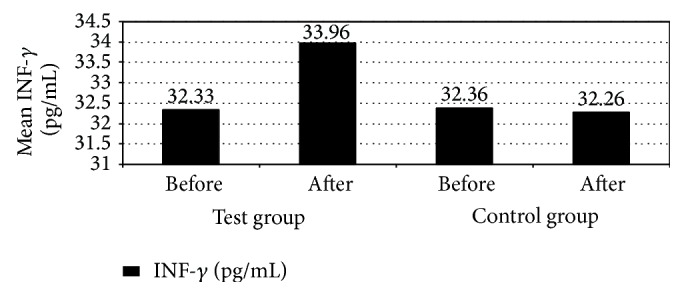
Mean interferon gamma (INF-*γ*) among the studied patients with colorectal cancer receiving chemotherapy (treated with recombinant human lactoferrin and not).

**Figure 3:**
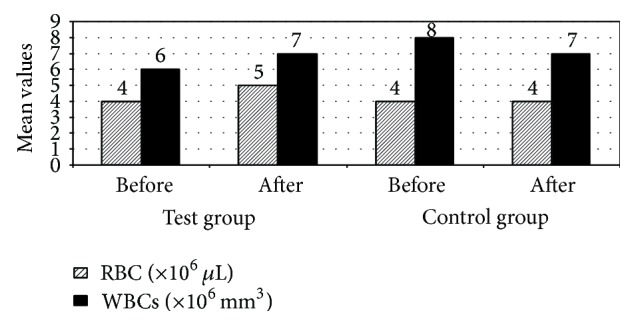
Mean values of RBCs and WBCs findings among the studied patients with colorectal cancer receiving chemotherapy (treated with recombinant human lactoferrin and not).

Patients in test group 3 months after treatment had significant increase in INF-*γ* serum levels compared to patients in the control group (*P* ≤ 0.05).  Figure 2 indicates that mean serum INF-*γ* increased from 32.33 pg/mL before treatment to 33.96 pg/mL after 3 months of treatment in patients in the test group compared to mean serum INF-*γ* of patients in the control group which was 32.36 pg/mL before treatment then 32.26 pg/mL after 3 months of treatment. This result may be due to modulation of cytokine production from leukocyte populations by lactoferrin [19]. Lactoferrin can increase* in vivo* and* in vitro* production of IL-12, a cytokine produced by antigen presenting cells (APCs) [19]. IL-12 functions to enhance production of IFN-*γ*, increase proliferation, and augment the cytotoxic activity of lymphocytes of the innate (NK cells) and adaptive (CD4^+^ and CD8^+^ T-cells) immune responses [19]. It is well known that colorectal cancer is like other solid malignancies which are infiltrated by various types of immune cells. Cells of the innate immune system, such as neutrophils, mast cells, natural killer (NK) cells, dendritic cells (DC), and tumor-associated macrophages, can be easily detected in these tumors [18]. So we can say that LF has a direct effect on inflammatory cells that participate in colorectal cancer pathogenesis. Also, WBCs count and neutrophil count had a significant increase in patients in the test group 3 months after treatment compared to patients in the control group (*P* ≤ 0.05).  Figure 3 indicates that mean WBCs count increased from 6 (∗10^3^ cell/*μ*L) before treatment to 7 (∗10^3^ cell/*μ*L) after 3 months of treatment in patients in test group compared to mean WBCs count of patients in the control group which decreased from 8 (∗10^3^ cell/*μ*L) before treatment to 7 (∗10^3^ cell/*μ*L) after 3 months of treatment. This result indicates that oral bLF enhances the immune system of colorectal cancer patients. As a result of this increase in WBCs and neutrophil count patients disease state may be improved, because the body immune system can fight the disease more efficiently compatible with some previous studies [20]. Also, results are nearly matched with those that were obtained in several clinical studies, where statistically significant increases were found between presupplementation levels and levels after 200 mg oral bLF supplementation in total T-cell activation, helper T-cell activation, cytotoxic T-cell activation, and hydrophilic antioxidant capacity [21]. These results support the proposal that oral supplements of bovine lactoferrin may be a useful adjunct toward modulation of immune activity, in particular T-cell activation and antioxidant status [21]. Lactoferrin is also known to exert changes on leukocytes of the innate immune system, through increasing natural killer (NK) cell activity, promoting function of neutrophils by enhancing phagocytic activity, modifying the production of reactive oxygen species, and activating macrophages through increasing cytokine and NO production and limiting intracellular pathogen proliferation [19]. The degranulation of neutrophils in response to inflammatory signals introduces lactoferrin into an environment that is populated with a mix of both innate leukocytes (macrophages, dendritic cells, and NK cells) and adaptive immune cells (T-cells and B-cells) [19]. The discovery of lactoferrin receptors on a wide variety of immune cells and their demonstrated capability to bind lactoferrin confirms the potential for this molecule to function in a manner to modulate and affect responses of both the innate and adaptive immune system [19].

**Figure 4:**
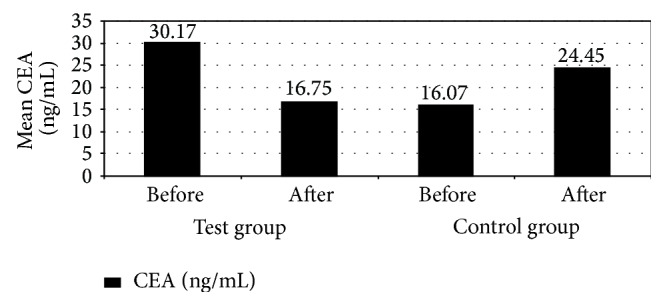
Mean carcinoembryonic antigen (CEA) and as a tumor marker among the studied patients with colorectal cancer receiving chemotherapy (treated with recombinant human lactoferrin and not).

A significant increase in serum GST enzyme in patients in the test group 3 months after treatment compared to patients in the control group (*P* ≤ 0.05) was observed.  Figure 1 indicates that the mean serum GST enzyme increased from 0.679 ng/mL before treatment to 0.886 ng/mL after 3 months of treatment in patients in the test group compared to mean serum GST enzyme of patients in the control group which did not change from 0.729 ng/mL before treatment to 0.729 ng/mL after 3 months of treatment. This may be due to regulation of the activities of phase II enzymes such as glutathione* S*-transferase [22]. The effect of antioxidants such as LF increase intracellular glutathione (GSH) levels in vascular endothelial cells by modulation of the GSH redox [23]. The serum GST enzyme has a detoxifying effect and so the increase in its serum level may help in colorectal cancer treatment [19]. Also there was a significant decrease in one of colorectal cancer tumor markers CEA in patients in test group 3 months after treatment in comparison with the patients in the control group (*P* ≤ 0.05).  Figure 4 indicates that mean serum CEA decreased from 30.17 ng/mL before treatment to 10.75 ng/mL after 3 months of treatment in test group compared to mean serum CEA of the control group which increased from 16.07 ng/mL before treatment to 24.45 ng/mL after 3 months of treatment. Also, this decrease may be resulted from improvement of the disease state as we mention that LF has an immunomodulatory property, which could play a major role in its antitumor activity [22]. Enhancement of an antitumor immunological response may restrict tumor growth. Many studies showed that LF elevates the number and increases the activity of T and B lymphocytes and NK cells, stimulates the release of a number of cytokines such as INF-*γ*, increases phagocytic activity and cytotoxicity of monocytes/macrophages, accelerates the maturation of T and B cells, and elevates the expression of several types of cellular receptors [22]. Apart from its immunomodulatory properties, LF exhibits direct antitumor activity, such as lytic, proapoptotic, antiproliferative, antiangiogenic, antioxidant activity, and chelating iron ions [22]. LF also, possesses chemopreventive properties, regulates the activity of phase I and II enzymes, which participate in the activation and detoxification of carcinogens, and regulates the composition of the intestinal microflora. In this way, it prevents the proliferation of tumors and their development at early stages of carcinogenesis [22]. These results are matched with results obtained in a former clinical trial made by Mai Abd El-Khalik in Menoufiya University to study the immunomodulatory and therapeutic effect of dietary lactoferrin in patients with colorectal cancer.

Now we can say that oral bLF may be a good adjuvant therapy for metastatic colorectal cancer patients due to its anti-inflammatory immunomodulatory effect that can reduce the chance of disease recurrence, infections, and inflammations so the oral bLF administration can enhance patient quality of life.

On the other hand, oral bLF administration decreased chemotherapy related side effects as it enhances both renal and hepatic function tests.

**Figure 5:**
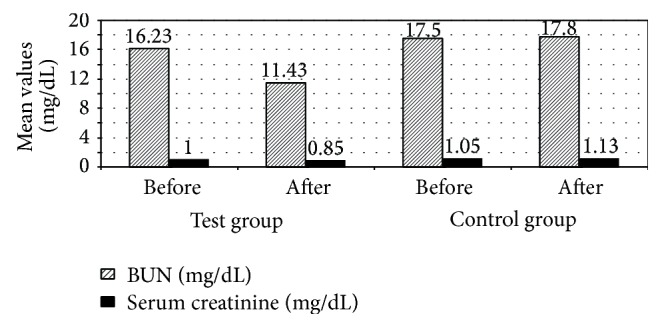
Mean blood urea nitrogen (BUN) and serum creatinine among the studied patients with colorectal cancer receiving chemotherapy (treated with recombinant human lactoferrin and not).

As mentioned in the results, there were significant decreases in BUN and serum creatinine in results of patients in the test group 3 months after treatment compared to patients in the control group (*P* ≤ 0.05).  Figure 5 indicates that mean serum BUN decreased from 16.23 mg/dL before treatment to 11.43 mg/dL after 3 months of treatment in patients in the test group compared to mean serum BUN of patients in the control group which changed from 17.5 mg/dL before treatment to 17.8 mg/dL after 3 months of treatment and indicates that mean serum creatinine decreased from 1 mg/dL before treatment to 0.85 mg/dL after 3 months of treatment in patients in the test group compared to mean serum creatinine of patients in the control group which changed from 1.05 mg/dL before treatment to 1.13 mg/dL after 3 months of treatment. This indicates that oral bLF improves renal functions and protects kidney from damage. This decrease in BUN and serum creatinine may be due to the antioxidant effect of LF as mentioned in a former preclinical study on rat model of ferric nitrilotriacetate- (Fe-NTA-) induced renal tubular oxidative injury. After an intraperitoneal administration of Fe-NTA for 4 and 24 h, bLF pretreatment suppressed elevation of serum creatinine and blood urea nitrogen levels. In addition, protective effects against renal oxidative tubular damage and maintenance of antioxidant enzyme activities in the bLF-pretreated group were observed [24].

**Figure 6:**
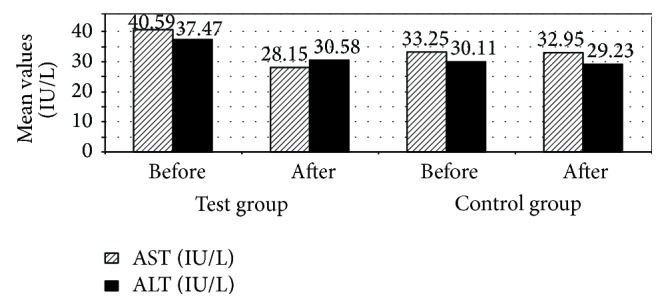
Mean aspartate aminotransferase (AST) and alanine aminotransferase (ALT) among the studied patients with colorectal cancer receiving chemotherapy (treated with recombinant human lactoferrin and not).

Also, there was a significant decrease in serum ALT and AST in patients in test group 3 months after treatment compared to patients in the control group (*P* ≤ 0.05).  Figure 6 indicates that mean serum AST decreased from 40.59 IU/L before treatment to 28.15 IU/L after 3 months of treatment in test group compared to mean serum AST of patients in control group which changed from 33.25 IU/L before treatment to 32.95 IU/L after 3 months of treatment and indicates that mean serum ALT decreased from 37.47 IU/L before treatment to 30.58 IU/L after 3 months of treatment in patients in test group compared to mean serum ALT of patients in control group which decreased from 30.11 IU/L before treatment to 29.23 IU/L after 3 months of treatment. Oral lactoferrin may increase liver functions and protect it from damage by reactive oxygen species since LF can function as an antioxidant, reducing intracellular levels of ROS [19]. It was hypothesized that lactoferrin could function to reduce oxidative stress-induced apoptosis [19]. Apoptosis is a programmed cell death [19]. In a former preclinical study carried on rats that aimed to compare the hepatoprotective effects of native bLF in relation to PEGylated Lactoferrin in a model of acute liver injury induced by D-galactosamine and lipopolysaccharide (GalN/LPS). Native bLf pretreatment was shown to suppress any increase in serum level of AST or ALT that was induced by GalN/LPS. Preadministration of 40k and 20k-PEG-bLf significantly suppressed the elevation of serum levels of AST and ALT induced by GalN/LPS [25].

Oral bLF administration enhanced anemia which is a very common chemotherapy related side effect; as results have shown, there were significant increases in RBCs count and serum Hb in patients in test group 3 months after treatment compared to patients in control group (*P* ≤ 0.05).  Figure 3 indicates that mean RBCs count increased from 4(∗10^6^/*μ*L) before treatment to 5(∗10^6^/*μ*L) cells after 3 months of treatment in patients in test group compared to mean RBCs count of patients in control group which did not change from 4(∗10^6^/*μ*L) cells before treatment to 4(∗10^6^/*μ*L) cells after 3 months of treatment. These results indicate that oral bLF could be used in treating anemia in metastatic colorectal cancer patients to improve their overall health state. These results are supported by a previous clinical trial in advanced cancer patients on chemotherapy; the results of this study show similar efficacy for oral lactoferrin and for I.V. iron, combined with rHuEPO, for the treatment of anemia in advanced cancer patients on chemotherapy [26]. The same results were also reported in another clinical trial on pregnant women 30 days after oral administration of bLf; hemoglobin and total serum iron levels increased to a greater extent than those observed in women treated orally for 30 days with ferrous sulfate, independent of the trimester of pregnancy [27]. Unlike ferrous sulfate, bLF did not result in any side effects [27]. Bovine LF restored both red and white peripheral blood cell numbers depleted by chemotherapy in a preclinical study, which indicate the ability of oral bLF in treating anemia [11].

There was a significant increase in platelets count in patients in test group after 3 months compared to control group (*P* ≤ 0.05).

Clinical parameters follow-up results indicate that oral bLF improved most of these parameters which indicates that patients' disease states became stable without any recurrence of colorectal tumor masses or appearance of any new changes which may be due to several physiological roles of LF: regulation of iron homeostasis, modulation host defense against infection and inflammation, regulation of cellular growth, differentiation, protection against cancer development, and metastasis [10]. These findings have suggested that LF has a great potential therapeutic use in cancer disease since it acts as a chemopreventive agent [10].

Oral mucositis is one of the most important clinical parameters that indicate therapeutic efficiency of oral bLF. Oral mucositis is one of the most common toxicities observed during radiotherapy and chemotherapy treatment for cancers. Mucositis results in sore mouth, altered taste sensation, pain, and dysphagia leading to malnutrition. If left untreated, oral mucositis leads to ulceration, orodental infection, bleeding, and discontinuation of effective radiotherapy or chemotherapy [28].

Patients in test group had less severed mucositis than patients in control group after every chemotherapy cycle; this result may be explained on the bases that pathogenesis of mucositis includes oxidative stress and releases reactive oxygen species (ROS). The latter could directly damage cells, tissues, and blood vessels with subsequent transcription factor activation [28]. Among transcription factors the nuclear factor-kB (NF-kB) appears to be the most prominent. It is activated by both radiation and chemotherapy and could upregulate genes that lead to the production of a group of proinflammatory cytokines, including tumor necrosis factor *α* (TNF-*α*). So anti-inflammatory agents, anti-infective agents, and reactive oxygen species inhibitors are used to treat oral mucositis [28].

As mentioned before, lactoferrin has protective effects that range from direct antimicrobial activities against a large panel of microorganisms, including bacteria, viruses, fungi, and parasites, to anti-inflammatory and anticancer activities [2] and has antioxidant effect [19] that can protect patients with metastatic colorectal cancer from recurrent infections and inflammations.

Another important parameter is infection recurrence; patients in test group after 3 months had a lesser rate of infection recurrence than patients in control group and had less severe symptoms of infection and fever, because lactoferrin plays an important role in immune regulation and defense mechanisms against bacteria, fungi, and viruses [3]. Lactoferrin's iron withholding ability is related to inhibition of microbial growth as well as to modulation of motility, aggregation, and biofilm formation of pathogenic bacteria. Independent of iron binding capability, lactoferrin interacts with microbial, viral, and cell surfaces, thus, inhibiting microbial and viral adhesion and entry into host cells [3]. Lactoferrin can be considered not only a primary defense factor against mucosal infections, but also a polyvalent regulator which interacts in viral infectious processes [3].

Results of patients in the test group indicate a significant effect of oral bLF 250 mg/day shown by significant improvement in mean percent of change of all parameters values after than before treatment among the studied patients with colorectal cancer receiving chemotherapy after 3 months (Tables 5 and 6), but we need to investigate effect of oral bLF on colorectal cancer patients for longer period than 3 months.

## 7. Conclusion

Oral bLF gave promising results in this clinical trial; it has the ability to improve symptoms of cancer in metastatic colorectal cancer patients, such as anemia, as it increased both RBCs count and Hb concentration. Also, LF decreased chemotherapy related side effects by protecting liver and kidney from toxicity and improving their function test values. Another very important chemotherapy related side effect is mucositis which improved by oral bLF ingestion and gave the chance to patients to swallow better with less pain and suffering. We can say that oral bLF has significant therapeutic effect on colorectal cancer patients after using for a long period of time. These may need a long term study.

## Figures and Tables

**Table 1:** Patients characteristics.

Number of male patients	20
Number of female patients	10
Number of smoker males patients	16
Age (years)	20–71
Body weight (kg)	54–98
Body height (cm)	146–178
Body surface area m²	1.5–2.3
Number of patients having hypertension	2
Number of patients having diabetes mellitus	2
Number of patients on warfarin	2
Number of patients having hepatitis C	1
Number of patients having history of bilharzias	1

**Table 2:** No significant differences between parameters values of patients in the test and control groups before the trial (results represented as mean ± SD values).

Parameter	Control mean ± SD	LF mean ± SD
BUN (mg/dL)	17.5 ± 8.04	16.23 ± 5.55
Serum creatinine (mg/dL)	1.05 ± 0.47	0.996 ± 0.524
AST (IU/L)	33.25 ± 18.4	40.59 ± 19.07
ALT (IU/L)	30.1 ± 16.19	37.46 ± 27.79
Serum LF (ng/mL)	0.186 ± 0.039	0.13 ± 0.018
Serum GST enzyme (ng/mL)	0.729 ± 0.073	0.679 ± 0.076
INF-*γ* (pg/mL)	32.36 ± 4.76	32.33 ± 4.65
WBCs (∗10^3^ cell/*µ*L)	7.55 ± 1.65	6.12 ± 1.97
Platelet (∗10^3^/mm^3)^	217.2 ± 88.76	207.6 ± 69.84
CEA (ng/mL)	16.07 ± 17.37	30.16 ± 50.06
RBCs (∗10^6^/*µ*L)	4.3 ± 0.41	4.4 ± 0.63
Neutrophil (%)	46.86 ± 7.21	45.46 ± 7.37
Hb (g/dL)	11.1 ± 2.1	11.09 ± 1.67

Note: ^*^significant difference *P* ≤ 0.05.

**Table 3:** Comparison between parameters values of patients in test and control groups after 3 months (results represented as mean ± SD values).

Parameter	Control mean ± SD	LF mean ± SD
BUN (mg/dL)	17.8 ± 6.49	11.43 ± 3.7^*^
Serum creatinine (mg/dL)	1.13 ± 0.398	0.851 ± 0.369
AST (IU/L)	32.94 ± 17.57	28.15 ± 11.83
ALT (IU/L)	29.22 ± 12.54	30.58 ± 18.32
Serum LF (ng/mL)	0.188 ± 0.035	0.282 ± 0.175
Serum GST enzyme (ng/mL)	0.72 ± 0.07	0.88 ± 0.08
INF-*γ* (pg/mL)	32.26 ± 4.81	33.96 ± 4.68^*^
WBCs (∗10^3^ cell/*µ*L )	7.41 ± 2.3	7.19 ± 1.65
Platelet (∗10^3^/mm^3^)	191.8 ± 78.46	244.06 ± 74.87
CEA (ng/mL)	24.44 ± 26.56	16.75 ± 28.32
RBCs (∗10^6^/*µ*L)	4.2 ± 0.46	4.9 ± 0.71
Neutrophil (%)	45.93 ± 5.1	57.33 ± 8.6
Hb (g/dL)	10.58 ± 1.6	13.06 ± 1.9

Note: ^*^significant difference *P* ≤ 0.05.

**Table 4:** Effect of oral administration of bLF on patients in test group after 3 months (results represented as mean ± SD values).

Parameter	Control mean ± SD	LF mean ± SD
BUN (mg/dL)	0.3 ± 2.9	4.8 ± 3.14^*^
Serum creatinine (mg/dL)	0.078 ± 0.22	0.14 ± 0.26^*^
AST (IU/L)	0.306 ± 7.29	12.44 ± 13.22^*^
ALT (IU/L)	0.88 ± 5.4	6.88 ± 11.27^*^
Serum LF (ng/mL)	0.002 ± 0.011	0.15 ± 0.16^*^
Serum GST enzyme (ng/mL)	0.000 ± 0.002	0.206 ± 0.089^*^
INF-*γ* (pg/mL)	0.1 ± 0.27	1.62 ± 0.66^*^
WBCs (∗10^3^ cell/*µ*L)	0.146 ± 0.727	1.06 ± 1.18^*^
Platelet (∗10^3^/mm^3^)	25.40 ± 39.06	36.46 ± 12.26^*^
CEA (ng/mL)	8.37 ± 23.91	13.41 ± 21.91^*^
RBCs (∗10^6^/*µ*L)	0.111 ± 0.222	0.514 ± 0.126^*^
Neutrophil (%)	0.93 ± 4.7	11.86 ± 1.45^*^
Hb (g/dL)	0.52 ± 1.2	1.97 ± 0.35^*^

Note: ^*^significant difference *P* ≤ 0.05.

**Table 5:** Mean percent of change of main parameters after than before treatment among the studied patients with colorectal cancer receiving chemotherapy (treated with recombinant human lactoferrin and not).

Parameters	Mean percent of change (%) among the studied patients with colorectal cancer receiving chemotherapy (*n* = 30)		*t*-test	*P*
	Group 1 (treated with lactoferrin) (*n* = 15)	Group 2 (not treated with lactoferrin) (*n* = 15)		
Serum lactoferrin (LF) (ng/mL):				
Range	9.73–414.71	↓8.31–9.09	4.380	0.001^*^
Mean ± SD	111.17 ± 96.69	1.65 ± 5.36		
Serum GST (ng/mg):				
Range	9.22–100.20	↓0.56–0.71	6.221	0.001^*^
Mean ± SD	31.83 ± 19.83	↓−0.26 ± 0.41		
Interferon gamma (INF-*γ*) (pg/mL):				
Range	1.21–10.62	↓2.57–0.73	9.027	0.001^*^
Mean ± SD	5.12 ± 2.18	↓−0.33 ± 0.84		

Note: ^*^significant (*P* ≤ 0.05).

**Table 6:** Mean percent of change of the remaining parameters after than before treatment among the studied patients with colorectal cancer receiving chemotherapy (treated with recombinant human lactoferrin and not).

Parameters	Mean percent of change (%) among the studied patients with colorectal cancer receiving chemotherapy (*n* = 30)		*Z*-test	*P*
	Group 1 (treated with lactoferrin) (*n* = 15)	Group 2 (not treated with lactoferrin) (*n* = 15)		
CEA (ng/mL):				
Range	↓98.02–↓20.83	↓89.53–400.00	3.791	0.001^*^
Mean ± SD	↓−47.45 ± 20.91	95.92 ± 144.93		
RBC (×10^6^/microL):				
Range	5.72–15.20	↓11.59–7.41	10.015	0.0001^*^
Mean ± SD	11.65 ± 2.15	↓−11.65 ± 2.15		
Hemoglobin (HB) (g/dL):				
Range	13.91–24.72	↓23.65–9.64	8.456	0.0001^*^
Mean ± SD	17.91 ± 2.76	↓−3.75 ± 9.53		
WBCs (×10^3^/mm^3^):				
Range	↓29.00–40.68	↓26.82–18.89	4.152	0.0001^*^
Mean ± SD	21.51 ± 18.27	↓−1.40 ± 11.09		
Platelets (×10^3^/mm^3^):				
Range	3.43–21.94	↓39.05–8.81	7.222	0.0001^*^
Mean ± SD	18.51 ± 4.56	↓−10.38 ± 14.81		
Neutrophil (%):				
Range	20.97–28.57	↓19.67–14.63	11.092	0.0001^*^
Mean ± SD	26.31 ± 2.11	↓−1.08 ± 9.33		
BUN (mg/dL):				
Range	↓53.00–↓16.67	↓19.44–57.89	5.858	0.0001^*^
Mean ± SD	↓−28.18 ± 10.47	7.92 ± 21.44		
Serum creatinine (mg/dL):				
Range	↓40.00–100	↓16.67–70.73	1.596	0.122
Mean ± SD	↓−5.24 ± 37.15	13.03 ± 24.20		
AST (IU/L):				
Range	↓62.07–↓17.60	↓53.85–89.87	3.757	0.001^*^
Mean ± SD	↓−27.50 ± 15.13	4.63 ± 29.46		
ALT (IU/L):				
Range	↓32.23–155.56	−23.19–28.71	0.755	0.457
Mean ± SD	↓−5.66 ± 46.83	3.97 ± 15.96		

Note: ^*^significant (*P* ≤ 0.05).
