# Corrigendum to “Study on the Therapeutic Benefit on Lactoferrin in Patients with Colorectal Cancer Receiving Chemotherapy”

**DOI:** 10.1155/2015/424603

**Published:** 2015-05-31

**Authors:** Tarek M. Moastafa, Alaa El-Din Elsayed El-Sissy, Gehan K. El-Saeed, Mai Salah El-Din Koura

**Affiliations:** ^1^Faculty of Pharmacy, Tanta University, Tanta, Egypt; ^2^Pharmacology Department, Faculty of Pharmacy, Tanta University, Tanta, Egypt; ^3^Faculty of Medicine, Menoufiya University, Shibin el-Kawm, Egypt; ^4^Hospital of Menoufiya University, Shibin el-Kawm, Egypt

Changes in the paper entitled “Study on the Therapeutic Benefit on Lactoferrin in Patients with Colorectal Cancer Receiving Chemotherapy” are as follows:

1. Title of Table 4 was changed.
2. Tables 5 and 6 became one table, currently numbered 5, containing updated information compared to the published paper.

## Figures and Tables

**Table 4:** Differences between parameters values before treatment and three months after treatment indicate improvement of all parameters after treatment among the studied patients in test group with colorectal cancer receiving chemotherapy and bLF (results represented as mean ± SD values).

Parameter	Control mean ± SD	LF mean ± SD
BUN (mg/dL)	0.3 ± 2.9	4.8 ± 3.14^*∗*^
Serum creatinine (mg/dL)	0.078 ± 0.22	0.078 ± 0.22 0.14 ± 0.26^*∗*^
AST (IU/L)	0.306 ± 7.29	12.44 ± 13.22^*∗*^
ALT (IU/L)	0.88 ± 5.4	6.88 ± 11.27^*∗*^
Serum LF (ng/mL)	0.002 ± 0.011	0.15 ± 0.16^*∗*^
Serum GST enzyme (ng/mL)	0.000 ± 0.002	0.206 ± 0.089^*∗*^
INF-*γ* (pg/mL)	0.1 ± 0.27	1.62 ± 0.66^*∗*^
WBCs (*∗*10^3^ cell/*μ*L)	0.146 ± 0.72	1.06 ± 1.18^*∗*^
Platelet (*∗*10^3^/mm^3^)	25.40 ± 39.06	36.46 ± 12.26^*∗*^
CEA (ng/mL)	8.37 ± 23.91	13.41 ± 21.91^*∗*^
RBCs (*∗*10^6^/*μ*L)	0.111 ± 0.222	0.514 ± 0.126^*∗*^
Neutrophil (%)	0.93 ± 4.7	11.86 ± 1.45^*∗*^
Hb (g/dL)	0.52 ± 1.2	1.97 ± 0.35^*∗*^

Note: ^*∗*^significant difference *P* ≤ 0.05.

**Table 5:** Mean percent of change of all parameters after compared to before treatment among the studied patients with colorectal cancer receiving chemotherapy (treated with recombinant human lactoferrin and not).

Parameter	LF mean ± SD	Control mean ± SD	*P*
Serum LF (%)	111.17% ± 96.69%	1.65% ± 5.36%	0.001^*∗*^
Serum GST enzyme (%)	31.83% ± 19.83%	↓ −0.26% ± 0.41%	0.001^*∗*^
INF-*γ* (%)	5.12% ± 2.18%	↓ −0.33% ± 0.84%	0.001^*∗*^
CEA (%)	↓ −47.45% ± 20.91%	95.92% ± 144.93%	0.001^*∗*^
BUN (%)	↓ −28.18% ± 10.47%	7.92% ± 21.44%	0.0001^*∗*^
Serum creatinine (%)	↓ −5.24% ± 37.15%	13.03% ± 24.20%	0.122
AST (%)	↓ −27.50% ± 15.13%	4.63% ± 29.46%	0.001^*∗*^
ALT (%)	↓ −5.66% ± 46.83%	3.97% ± 15.96%	0.457
RBCs (%)	11.65% ± 2.15%	↓ −2.56% ± 5.06%	0.0001^*∗*^
Hb (%)	17.91% ± 2.76%	↓ −3.75% ± 9.53%	0.0001^*∗*^
WBCs (%)	21.51% ± 18.27%	↓ −1.40% ± 11.09%	0.0001^*∗*^
Platelet (%)	18.51% ± 4.56%	↓ −10.38% ± 14.81%	0.0001^*∗*^
Neutrophil (%)	26.31% ± 2.11%	↓ −1.08% ± 9.33%	0.0001^*∗*^

Note: ^*∗*^significant (*P* ≤ 0.05).

